# Integrative Taxonomy of *Nitraria* (Nitrariaceae), Description of the New Enigmatic Species and Key to All Currently Known Species

**DOI:** 10.3390/plants12030593

**Published:** 2023-01-29

**Authors:** Evgeny V. Banaev, Maria A. Tomoshevich, Sofia A. Khozyaykina, Anna A. Erst, Andrey S. Erst

**Affiliations:** Central Siberian Botanical Garden, Siberian Branch of Russian Academy of Sciences, Zolotodolinskaya Str. 101, Novosibirsk 630090, Russia

**Keywords:** integrative taxonomy, *Nitraria*, morphology, phylogeny, palynology, biochemistry, DNA, ITS, trnH–psbA, HPLC-MS

## Abstract

A new species, *Nitraria iliensis* sp. nov., is described from the Ili basin, Almaty region, Kazakhstan. It belongs to section *Nitraria ser. Sibiricae* and is morphologically similar to *N. sibirica* Pall. An integrative taxonomic approach based on molecular, biochemical and morphological analyses, along with palynological data, was used to delimit this new species. The studied species of the genus are illustrated, and photographs of authentic specimens of the new species, as well as a distribution map of the new species and segregate taxa, are provided. Morphological characters were investigated, more important traits for identification were found, and a new key to distinguish between all species of the genus was prepared.

## 1. Introduction

The genus *Nitraria* L. belongs to the monotypic family Nitrariaceae Bercht & J. Pres; this genus was previously assigned to the family Zygophyllaceae R. Br. [1,2]. It includes 9–11 species restricted to steppe, semi-desert and desert regions of Asia, North Africa, South-Eastern Europe (Romania), Papua New Guinea and Australia [3,4]. GBIF, Kew, Plant list, and IPNI databases list nine species: *N. tangutorum* Bobrov, *N. sphaerocarpa* Maxim., *N. sibirica* Pall., *N. schoberi* L., *N. komarovii* Iljin & Lava ex Bobrov, *N. roborowskii* Kom., *N. pamirica* L.I. Vassiljeva, *N. billardierei* DC., and *N. retusa* (Forsk.) Aschers.

The first data on *Nitraria* were reported by G. Schober, a famous scientist who explored the Volga region and the Caucasus of the Russian state in 1717–1720 [5]. K. Linnaeus grew plants from seeds collected by G. Schober in the lower reaches of the Volga river. These plants were later identified as the *N. schoberi* lectotype [6].

Later, P.S. Pallas [7] identified specimens of *Nitraria* growing in the vicinity of Astrakhan (lower reaches of the Volga river) as *N. schoberi* var. *caspica* and specimens from Siberia (according to the collections of G.V. Steller) as *N. sibirica*.

By the end of the 19th century, three more species of *Nitraria* had been described: in 1828, *N. billardierei* DC. from Australia; in 1876, *N. retusa* from North Africa; and in 1883, *N. sphaerocarpa* from Central Asia (Khami Gobi).

R.E. Trautvetter made an important contribution to the future description of another species from the eastern coast of the Caspian Sea near the town of Krasnovodsk (now known as Turkmenbashi, Turkmenistan) [8]. He distinguished this plant from *N. schoberi* by its long and narrow leaves gradually tapering toward the base. At the time, he named the taxon *N. schoberi* var. *polygama* Trautv. For the first time, the same plant was mentioned under a different name (*N. komarovii*) in the journal Priroda by M.M. Il’in [9] but without a legitimate diagnosis: “*N. komarovii* Iljin et Lava., sp. nov. (typus Krasnovodsk, littora maris Caspii, 22 October 1900, leg, Freyn)”. N.K. Kovtonyuk, M.A. Tomoshevich, and E.V. Banaev [10] conducted a detailed study to choose a lectotype, *N. komarovii* Iljin & Lava ex Bobrov, which was identified as a specimen of this plant from the herbarium of V.L. Komarov Botanical Institute, RAS: LE00050757. This specimen had been identified in 1960 by V.P. Bochantsev as a lectotype of *N. komarovii* but was not published properly.

The first major analysis of the genus *Nitraria* was carried out by V.L. Komarov [5]. He noted substantial variation of the morphological characters of *N. schoberi* vegetative organs without a clear-cut hiatus across the geographic range. In addition, he described another new species: *N. roborowskii*. According to his data, the plant from the Cherchen oasis (Kashgar) differed sharply from known specimens of *N. schoberi* in larger leaves, inflorescences, and drupes.

It should be pointed out that the taxonomic position and distribution of *N. roborowskii* is the subject of debate among researchers [11,12,13]. In particular, E.G. Bobrov [14] believed that if only quantitative traits (gigantism) are taken into account, *N. roborowskii* should be regarded not as a species but as an ecological form: *N. sibirica* f. *majus*.

The most complete review of *Nitraria* systematics can be credited to E.G. Bobrov [14,15]. He divided the genus into two sections (sect. *Tridentatae* and sect. *Nitraria*) and described two new species: *N. tangutorum* (from Tsaidam, China) and *N. praevisa* (from the Alashan Gobi, China).

The tenth species of *Nitraria* described by L.I. Vasilieva [16] grows at an altitude of ~4000 m a.s.l. in the Eastern Pamirs: *N. pamirica*. According to that author’s description, the new species and closely related *N. sibirica* and *N. schoberi* differ mainly in an elongated conical stone with a reticulate-veined and irregularly lacunous surface, whereas in the two species, the stone surface is free of veins and contains only a few, rounded, deeper dimples. Furthermore, according to L.I. Vasilieva, this new species differs from *N. schoberi* in shape, leaf size, and bush habitus, and from *N. sibirica* in a larger cherry (not black) drupe, inflorescence axes pubescent with adpressed hairs, and in the pistil that is almost twofold shorter than the petals. 

Recent studies have revealed the morphological and genetic diversity, phylogeny, and putative origin of some species of the genus *Nitraria* [17,18,19,20,21,22]. Along with species polymorphism, there are data on natural hybridization between species of the genus *Nitraria* [23].

For the studied region (Siberia, Crimea, Kazakhstan, and Tajikistan), only three *Nitraria* species (*N. schoberi*, *N. sibirica*, *N. pamirica*) are indicated in the literature [24,25,26,27,28,29,30]. Recently, another species, *N. komarovii*, has been recorded in the territory of Kazakhstan in the Balkhash-Alakol basin [31].

Recent studies of plants of the genus *Nitraria* in the territory of Kazakhstan have revealed a high genetic and morphological polymorphism of *N. sibirica*. Analysis of the sequence of the ITS of the nuclear ribosomal DNA identified two main *N. sibirica* ribotypes [32,33]. The heterogeneity of *N. sibirica* is also supported by data on composition and content of phenolic compounds in plants from Siberian and Kazakh populations [34] and the presence of morphological differences in plants from these populations [4,35].

The aim of the study was to comprehensively investigate the morphological (including palynological), molecular, genetic and biochemical traits of *N. sibirica*, *N. schoberi*, *N. komarovii*, and *N. pamirica* to confirm the possible taxonomic heterogeneity of the species. The relationship between these species and the new species described and referred to as *Nitraria iliensis* Banaev&Tomoshevich, sp. nov. was analyzed.

## 2. Results and Discussion

### 2.1. Morphological Analysis

**Figure 1:**
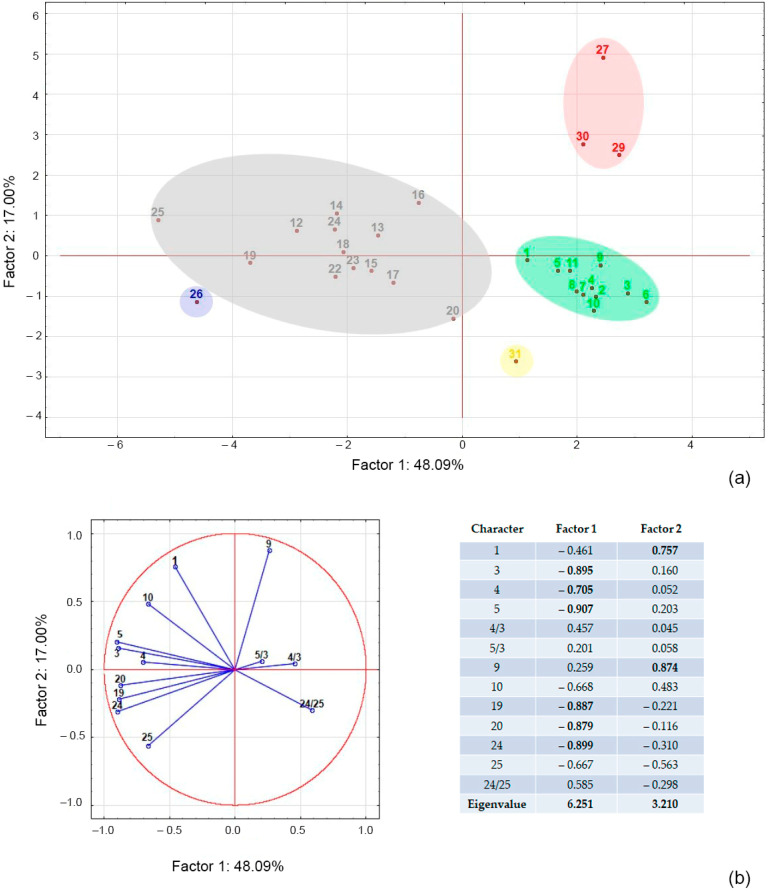
Principal component analysis (PCA) plot of various morphological characters of *Nitraria* species. (**a**) Scatter plot of various populations represented in two major principal component axes. *N. iliensis* (red), *N. sibirica* (green), *N. schoberi* (grey), *N. komarovii* (blue) and *N. pamirica* (yellow). (**b**) Grouping of the variables in two principal components. The most relevant characters of each principal component are shaded. See Table 1 for character number, Table 2 for specimen number.

We compared 26 characters to distinguish between *N. sibirica*, *N. schoberi*, *N. komarovii*, *N. pamirica*, and *N. iliensis* (Table 1). Principal component analysis showed that the first two principal components accounted for 65.1% of the variance of the entire array of parameter data and exhibited the best species discrimination (Figure 1a). It was found that in terms of the first factor, which brought about 48% of the variance, all the studied populations (species) were divided into two groups; the most relevant characters were leaf length, leaf width, distance from the base to the widest point of the leaf blade, fruit length, fruit width, and stone length (Table 1, Figure 1b). The populations of the new species were clearly separated in the plane of the second factor from the populations of *N. sibirica* and *N. pamirica*; the most significant characters were height of bush and number of flowers per inflorescence (Table 1, Figure 1b).

Analysis of variance (ANOVA, LSD test) showed significant differences between *N. iliensis* and *N. sibirica* closest to it in leaf blade length, *p* = 0.0008; number of flowers per inflorescence, *p* = 0.0031; drupe parameters (length *p* = 0.0382; width *p* = 0.0262) and stone parameters (width *p* = 0.0001; area *p* = 0.0002; perimeter *p* = 0.0273).

At the same time, the LSD test did not reveal significant differences (*p* ≤ 0.05) between populations within *N. sibirica* (11 populations) and *N. iliensis* (3 populations), which confirms the integrity and independence of these taxa.

Numerous researchers indicate indistinct morphological isolation of species of the genus *Nitraria* [14,15,16,32]. Some researchers [36,37] report the difficulty in identifying *Nitraria* species among herbarium specimens from West Siberia, as well as the need for studies of *Nitraria* species in natural populations. We previously showed that morphological characters of the species *N. sibirica* were not uniform across its distribution area [21]. In this study, for the first time, we performed a comprehensive analysis of the morphological characters of five species of the genus *Nitraria* from 31 populations, including a new species *N. iliensis*, and identified the most relevant diagnostic characters in the genus.

### 2.2. Palynological Analysis

**Figure 2:**
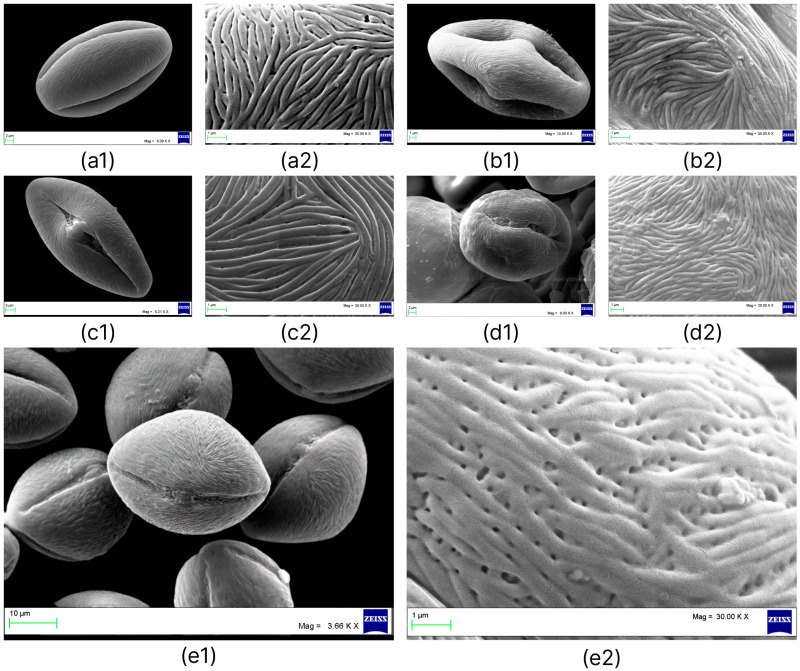
SEM analysis of *Nitraria* pollen grains: *N. sibirica* (**a1**,**a2**); *N*. *komarovii* (**b1**,**b2**); *N*. *shoberi* (**c1**,**c2**); *N*. *pamirica* (**d1**,**d2**); *N*. *iliensis* (**e1**,**e2**); **1**—equatorial view, **2**—details of the exine surface.

The main features of the investigated pollen grains of *Nitraria* were summarized (Table 3) and presented with scanning electron microscopy (SEM) micrographs (Figure 2). The *Nitraria* species analyzed here had medium pollen grains and varied from subprolate to prolate shape. P/E ratios of pollen grains showed that the highest P/E values belong to *N. sibirica* (1.70–1.97), which are of a prolate shape. The lowest was detected in *N. pamirica* (1.22), which are characterized by a subprolate pollen grains.

Pollen grains of *N. iliensis* are medium in size and are radially symmetrical. The mean length of the polar axes of them is 33.82 (28.71–37.29) μm, and the mean length of the equatorial axes is 25.81 (21.10–27.08) μm. The mean P/E ratio of the pollen is 1.32 (1.10–1.53). They are subprolate or prolate in equatorial view and triangular convex or pseudo-hexagonal, circular in polar view and monad, isopolar.

The aperture is tricolporate. Ectoaperture—colpus, almost as long as the polar axis, open (4/5 of polar axis), straight, narrow, occasionally constricted at equator with ends acute; polar area asymmetric. Margin observed in polar view, costae colpi and fastigium conspicuous in equatorial view. Endoaperture—porus, conspicuous, lalongate, elliptic to rhombic in shape. Exine—tectate, exine slightly thicker in polar areas in relation to equatorial region; nexine is thicker than sexine. The surface ornamentation is striate and perforated. Striae are relatively loose, packed in the mesocolpia, and short (Figure 2e1,e2).

The specimens of *N. iliensis* have a longer equatorial axis and a shorter polar axis compared with *N. sibirica* specimens; they exhibit a different striate–perforate ornamentation of pollen grains. The pollen morphology of *Nitraria* and *Peganum* is of a distinctly phylogenetic structure. Ancient species such as *N. sphaerocarpa* and *N. retusa* are considered to have pollen with a polar axis shorter than that in later divergent taxa, and a perforated exine [3,38]. Previously, we showed that striate–perforate ornamentation is typical only of the populations Basshi, Taskarasu, Karatal of *N. sibirica* (now *N. iliensis*) and it does not occur in the other four studied species of the genus *Nitraria* [4].

### 2.3. Molecular Analysis

**Figure 3:**
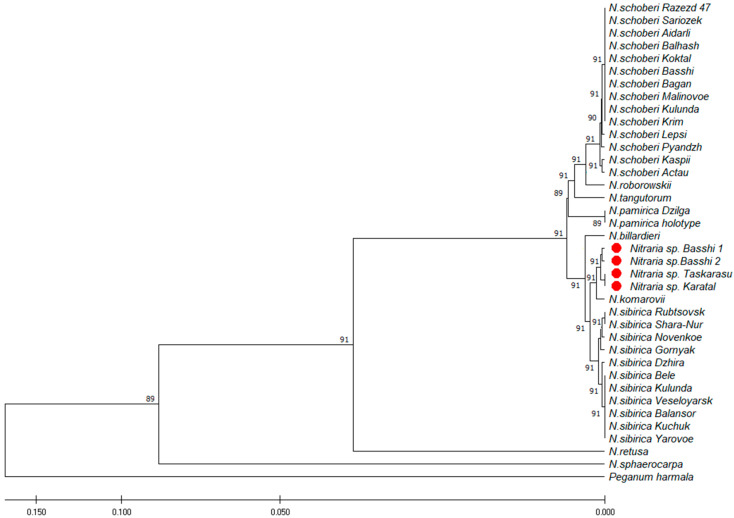
Phylogenetic tree inferred from the combined ITS and cpDNA data using the UPGMA method. The number near branches are bootstrap values (BS > 50%). Red symbols—*N. iliensis*.

**Figure 4:**
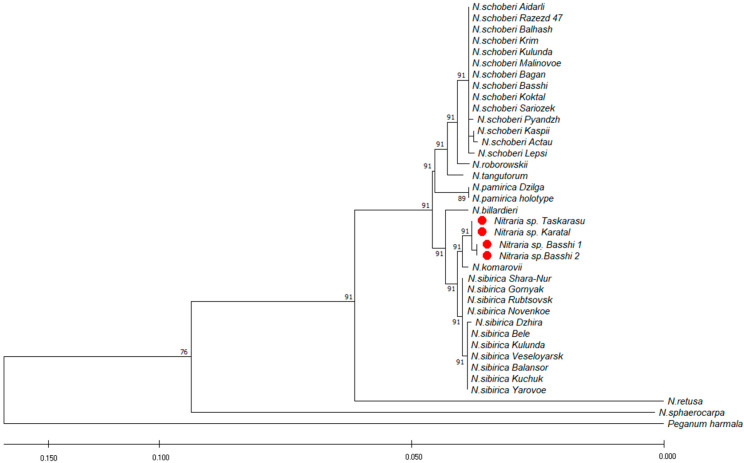
Phylogenetic tree inferred from the combined ITS and cpDNA data using the ML method. The number near branches are bootstrap values (BS > 50%). Red symbols—*N. iliensis*.

Phylogenetic trees constructed using UPGMA and ML were congruent. The phylogenetic tree (Figure 3 and Figure 4) showed that the new species (specimens of *Nitraria* sp.) is sister to *N. sibirica.* The specimen of *N. komarovii* included in this clade, the sequences of which were taken from the Genbank (KP087774; KP087766), is subject to debate (2.5.8. Notes). The monophyly of the new species, *N. sibirica* and *N. schoberi,* was supported.

The molecular approach is now becoming a common aspect of plant research at various taxonomic levels. Non-encoded regions of internal transcribed spacers (ITS) of nuclear ribosomal DNA genes are the most promising molecular markers for plant taxa identification [39]. Analysis of the sequence polymorphism of the internal transcribed spacers (ITS1, ITS2) revealed the Siberian and the Kazakh *N. sibirica* ribotypes [32,33]. Complete sequences of the chloroplast genomes reported for *N. sibirica* [40], *N. tangutorum* and *N. roborowskii* [41] determine the phylogenetic position of the species and the entire family Nitrariaceae in the Sapindales clade.

ISSR analysis revealed high interpopulation differentiation in *N. sibirica* in 22 natural populations (Russia and Republic of Kazakhstan) [22]. The authors report that the maximum genetic differences were recorded in Kazakhstani *Nitraria* specimens from the Ili basin. ISSR analysis of two marginal populations of *N. schoberi* from Romania, in contrast, showed low interpopulation diversity (He ≈ 0.2), which may be due to founder effects since populations most likely originated from a limited number of ancestral individuals [42].

### 2.4. HPLC-MS Analysis

Secondary metabolites of plants of the genus *Nitraria* are mainly represented by alkaloids and flavonoids [43]. Saleh et al. [44] highlighted the relationship between the composition of these compounds and the phylogeny of taxa of the Zygophyllaceae family. Literature analysis suggests the presence of specificity of individual components of secondary metabolites in species of the genus *Nitraria* [45,46,47,48,49]. A number of papers report on the specificity of phenolcarboxylic acids and flavonoids isolated from the leaves of *N. tangutorum*; therefore, they can be used in the taxonomy of the genus [50]. N. Barbhan et al. [51] showed that the chemical composition of the above-ground part of *N. retusa* differs from that of other species of the genus *Nitraria*.

Our phytochemical studies of *N. sibirica*, *N. schoberi*, *N. komarovii*, and *N. pamirica* from 58 populations of Russia, Kazakhstan, and Tajikistan performed using HPLC showed that the species differ in the composition and content of phenolic compounds [52,53]. A total of 27 phenolic compounds were identified. The maximum number (16–18 compounds) was found in the leaves of *N. sibirica*. *Nitraria* specimens from the populations of the Almaty region, Kazakhstan, lack individual phenolic compounds found in all populations of *N. sibirica*. The phenolic composition of *N. schoberi* is weaker compared with that of *N. sibirica*. Plants of this species mainly contain not more than 14 compounds. In the leaves of *N. pamirica*, 12 compounds were found, and not less than 6–8 compounds were identified in the leaves of *N. komarovii*.

**Figure 5:**
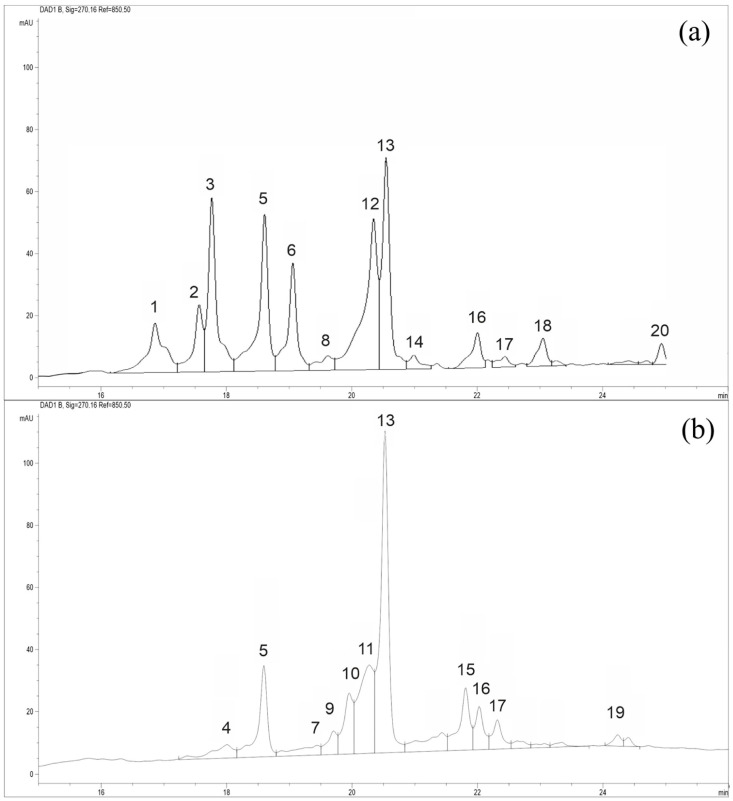
HPLC-MS chromatograms of 70% water–ethanol extracts *Nitraria* leaves: (**a**) *N. sibirica*, (**b**) *N. iliensis*. The abscissa is the retention time, t, min; ordinate—optical density, e.o.p., mAU; **1**—rutin; **6**—quercitin; **13**—hyperoside; **18**—baicalin; **20**—dihydroxymethoxyflavone; **2**–**5**, **7**–**12**, **1**4–**17**, **19**—unidentified compounds.

HPLC-MS analysis of *N. sibirica* and *N. iliensis* (Figure 5) showed a significant difference in the phenolic profiles of these species. The *N. iliensis* specimen contains a smaller number of phenolic compounds compared with *N. sibirica* (**11** and **13** compounds, respectively). Only four compounds are common to both species (hyperoside, compounds **5**,**16**,**17**); hyperoside is inherent in all studied species. Compound **5**, and minor compounds **16** and **17** were not detected in *N. schoberi*, *N. komarovii*, and *N. pamirica*.

### 2.5. Taxonomy

A comprehensive analysis of the obtained data distinguished a new species and showed its uniqueness in comparison with four related species growing in the study area: *N. sibirica*, *N. komarovii*, *N. pamirica*, and *N. schoberi*.

*Nitraria iliensis* Banaev&Tomoshevich, sp.nov.

**Figure 6:**
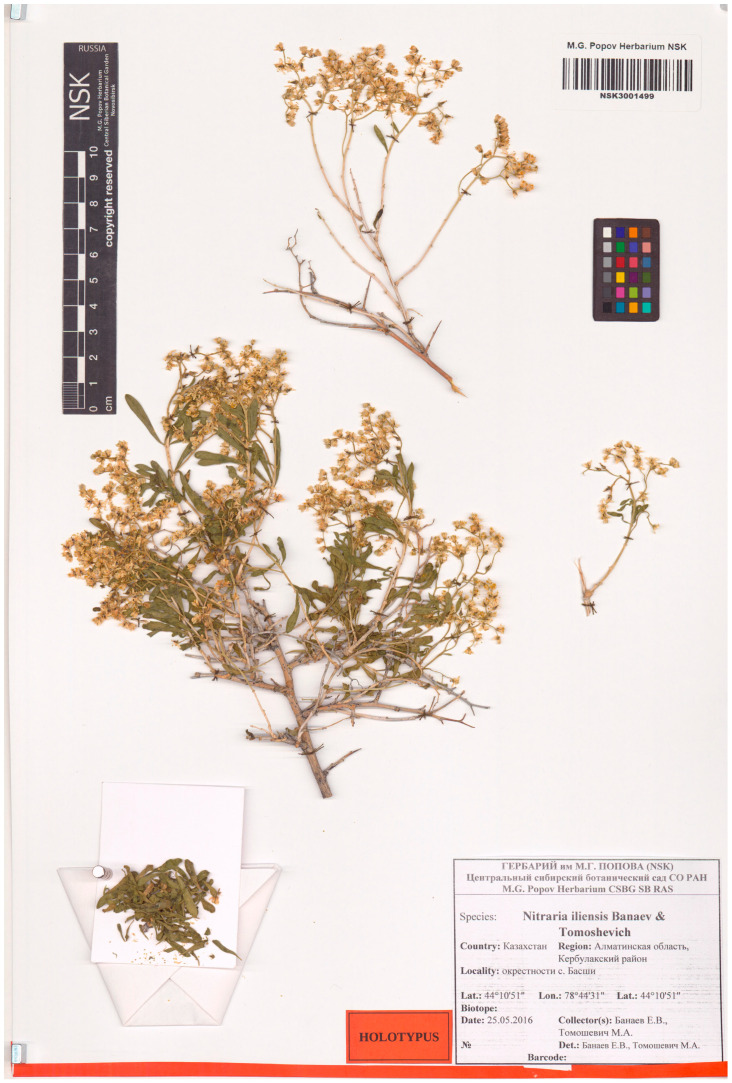
Holotype of *N. iliensis* Banaev & Tomoshevich, sp. nov. (NSK 3001499).

Type. Republic of Kazakhstan, Almaty region, vicinity of Basshi village, 44°10′51′′ N, 78°44′31′′ E, 1021 m a.s.l., 25 May 2016, *E. V. Banaev & M. A. Tomoshevich* (holotype, NSK3001499) (Figure 6).

**Figure 7:**
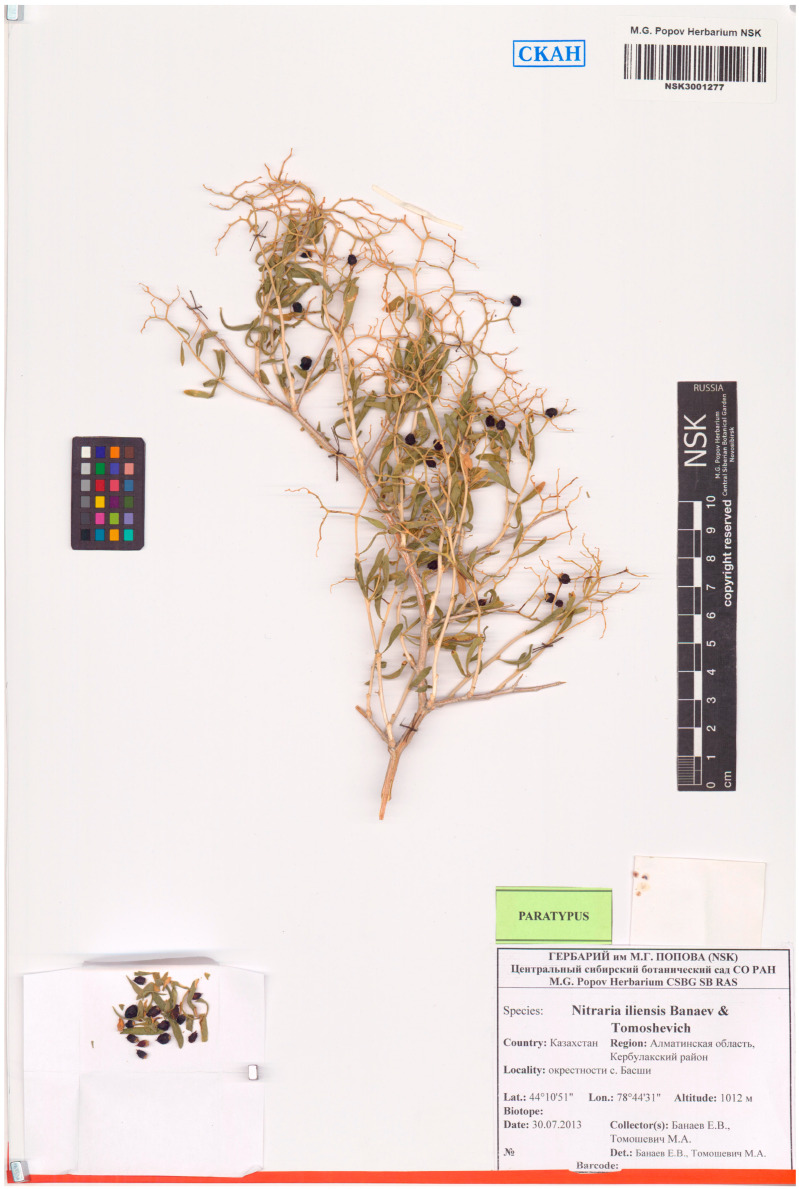
Paratype of *N. iliensis* Banaev & Tomoshevich, sp. nov. (NSK 3001277).

Paratype Republic of Kazakhstan, Almaty region, vicinity of Basshi village, 44°10′51′′ N, 78°44′31′′ E, 1021 m a.s.l., 30 July 2013, *E. V. Banaev & M. A. Tomoshevich* (NSK3001277) (Figure 7).

#### 2.5.1. Description

Bushes tend to be 0.6–1.8 m high, densely branched from the base, with slightly arched shoots sticking out in the center, and multispinous. The branches are bare with ash-gray, cracked bark; one-year-old shoots are yellowish, shiny, pubescent. The leaves (12) measure 14–17 (20) × 2–3 mm, are oblanceolate, gradually tapering towards the base, acute or obtuse at the apex, entire, green, fleshy. All leaves are pubescent on both sides. The ultimate inflorescence scorpioid cyme, with peduncle 5.5–15 cm; flowers 40–80. Flowers hermaphrodite, typically pentamerous. Peduncles and inflorescence axes slightly hairy. Resistant calyx up to 2.5 mm, fleshy, pubescent. Petals white, oblong-ovate, 2.8–3.9 × 1.5–2.6 mm, concave with incurved margins, claws short. The fruit is a fleshy black drupe, with black-green sap; oval or spherical, 4–6 mm long, 4–4.5 mm in diameter; finely pubescent; edible and salty-bitter. The sap of ripe berries stains white paper black–green. The stone is dark, reddish brown, narrow ovoid with a narrow pointed apex, 3.5–5 mm long, 2–2.6 mm in diameter.

#### 2.5.2. Affinity

The new species *N. iliensis* belongs to the sect. *Nitraria* ser. *Sibiricae* Bobrov [14], which is evidenced by the results of molecular phylogenetic analysis. Differences between the five species studied in Russia, Tajikistan, and Kazakhstan are summarized in Table 1.

**Figure 8:**
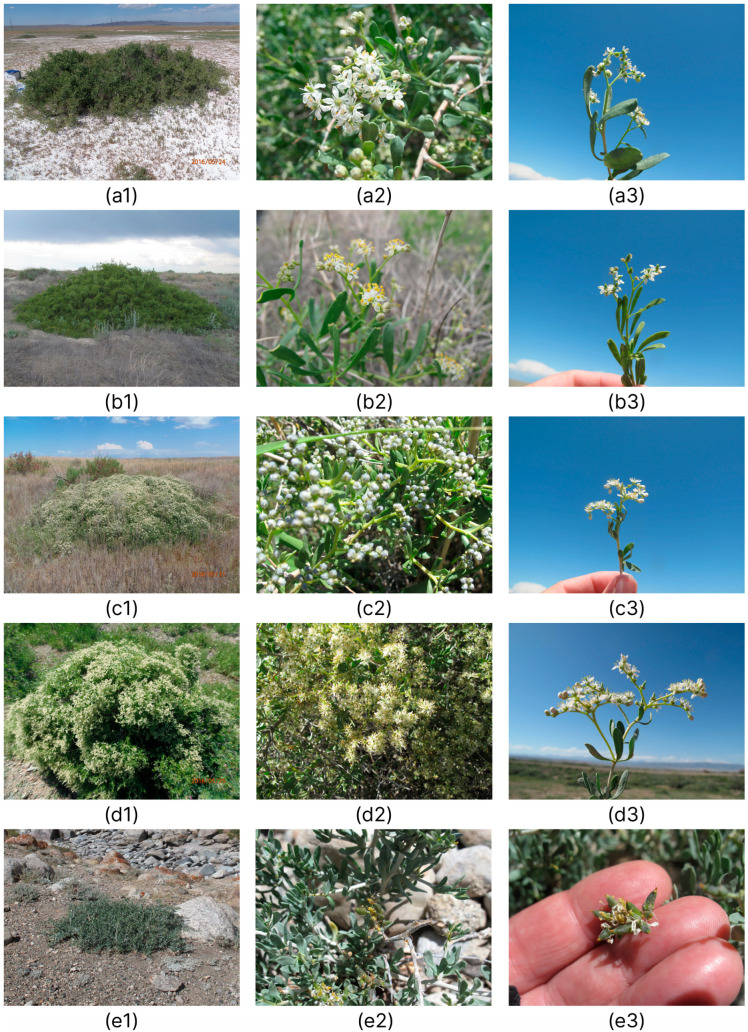
Morphological differences amongst (**a1**–**a3**) *N. schoberi*, (**b1**–**b3**) *N. komarovii*, (**c1**–**c3**) *N. sibirica*, (**d1**–**d3**) *N. iliensis,* and (**e1**–**e3**) *N. pamirica* at the flowering stage, 1—habit, 2—flowers, 3—inflorescence. Photo by Banaev E.V.

*Nitraria iliensis* is morphologically similar to *N. sibirica* (Figure 8) in spreading-branching, dense bush habit, oblanceolate leaf shape, flower shape and size, globate or oval black drupe.

**Figure 9:**
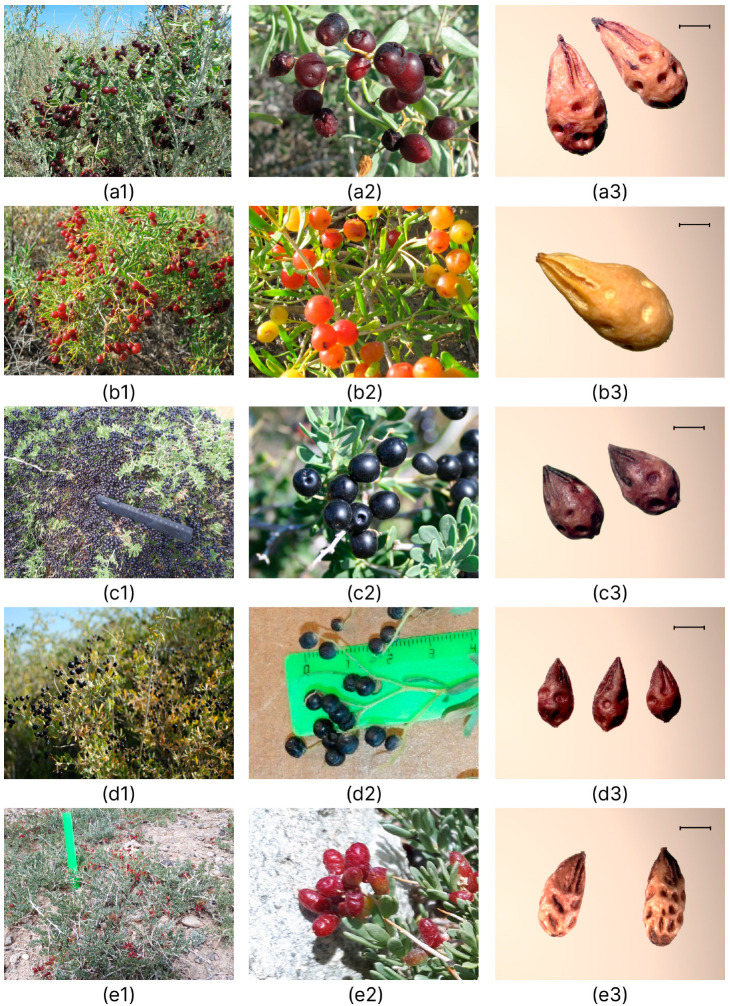
Morphological differences amongst (**a1**–**a3**) *N. schoberi*, (**b1**–**b3**) *N. komarovii*, (**c1**–**c3**) *N. sibirica*, (**d1**–**d3**) *N. iliensis* and (**e1**–**e3**) *N. pamirica* at fruiting stage, 1—habit, 2—fruit, 3—stone. Photo by Banaev E.V.

The new species differs from other related species in the height of the bush (0.6–1.8 m), size and color of the leaf blade, showing a smaller size of the fruit and stone, and a larger number of flowers per inflorescence (Table 1, Figure 8 and Figure 9).

#### 2.5.3. Phenology

Flowering: late May–early June, 5–7 days later than that in *N. schoberi*, but 2–3 days earlier compared with *N. sibirica*. Fruiting: end of July–beginning of August.

#### 2.5.4. Distribution

*Nitraria iliensis* is confined to the Almaty region, Republic of Kazakhstan, the Ili basin.

#### 2.5.5. Habitat and Ecology

*Nitraria iliensis* grows at an altitude of 400–1000 m a.s.l., in halophytic cenoses including *Tamarix*, *Halimodendron*, *Nitraria schoberi*, *Artemisia*, *Achnatherum*, *Halocnemum*, and *Swaeda*.

#### 2.5.6. Etymology

The specific epithet of the new species comes from the type locality, Ili basin, Almaty region, Republic of Kazakhstan.

#### 2.5.7. Additional Specimens Examined

Republic of Kazakhstan, Almaty region, Karatalskii district, vicinity of Ushtobe city, on the terrace of the Karatal river, 45°21.995′ N, 77°55.048′ E, 29 May 2016, E.V. Banaev & M.A. Tomoshevich, 3000922 (NSK); Republic of Kazakhstan, Almaty region, Karatalskii district, vicinity of Ushtobe city, on the terrace of the Karatal river, 45°21.995′ N, 77°55.048′ E, 15 August 2017, E.V. Banaev & M.A. Tomoshevich, 3001272 (NSK); Republic of Kazakhstan, Almaty region, vicinity of Taskarasu village, 43°46′54.60′′ N, 79°27′16.56′′ E, 26 May 2016, E.V. Banaev & M.A. Tomoshevich, 3001244 (NSK).

#### 2.5.8. Notes

The genebank contains sequences of nuclear and plastid DNA fragments isolated from voucher P. Farse, 13 May 1964, Afghanistan. This voucher specimen is stored in the herbarium of the Institute of Botany, Chinese Academy of Sciences (PE) and referred to as *N. komarovii*. However, *N. komarovii* is exclusively littoral and grows on the sands along the coasts of large lakes. E.G. Bobrov [14] considered it the youngest of all known species associated with the history of formation of the Caspian basin. Previously, it was only known from three habitats: the Krasnovodsk Peninsula, in vicinity of Turkmenbashi city (Turkmenistan), the Apsheron peninsula (Azerbaijan), and the mouth of the Volga river. We have identified one more locality for *N. komarovii* [31] on the coast of the lake Balkhash. Therefore, the presence of *N. komarovii* in the flora of Afghanistan is debatable.

A similar situation can be observed for *N. komarovii* recorded from the flora of Iran. H. Akhani [54] reports the absence of *N. komarovii* in the flora of Iran, and Iranian records of *N. komarovii* Iljin & Lava [55] are referred to *Atraphaxis suaedifolia* Jaub. & Spach (Polygonaceae).

It should be noted that data on the genus *Nitraria* in various databases are currently controversial. For example, in the Kew, Plant list, and IPNI databases “https://powo.science.kew.org/taxon/urn:lsid:ipni.org:names:873338-1 (accessed on 22 December 2022)”, *N. praevisa* is a synonym for *N. roborowskii* according to E.G. Bobrov [14]. However, E.G. Bobrov is the author of the species *N. praevisa*, which he described in this work in 1965. In the key, he included *N. praevisa* along with *N. roborowskii*. 

#### 2.5.9. Key to *Nitraria* Species

All characters in the key of species of the genus *Nitraria* are described from the studied material from natural populations and authentic specimens stored in the LE herbarium.
1. Drupe juicy, oval or spherical; stone ovoid or conical.............................................................2- Drupe dry, swollen, spherical up to 1 cm in diameter, with a membranous swollen pubescent shell; narrow stone, almost fusiform, 0.8 × 0.2–0.3 cm. Thorny bush, prostrate, up to 20–40 cm high, with curved branches, pillow-shaped. Branches are grayish-white in color. Leaves narrow, linear-lanceolate, 20–40 × 1–3 mm. Inflorescence shoot short, up to 5–10 cm. Flowers white, small, up to 0.5 cm....................................................*N. sphaerocarpa* Maxim2. Leaves obovate, oblanceolate or linear, entire above, very rarely 1–2-toothed; stipules membranous, deciduous....3- Leaves spatulate or cuneiform, entire above or 3–5 crenate-toothed; stipules present........*N. retusa* (Forsk.) Aschers3. Leaves entire, blunt at the apex, rarely pointed...................................................................4- Leaves are the largest of all species of the genus, 25–46 × 6–9 mm, either entire or 1–2-toothed at the apex (on vegetative shoots); drupe large, round, juicy, pale pink to maroon in color, stone 10–12 mm long, 3.5–4.5 mm wide, ovoid pointed to apex, barely pointed or almost blunt; bush 1–1.5 m high................................*N. roborowskii* Kom.4. Bush above 0.5 m, spreading-branching..........................................................................5- Bush 0.1–0.3 m high, spreading, dense; leaves green, matte, oblanceolate with a pointed apex; number of flowers in the inflorescence 9–15; petals white, oblong-oval with short claws; drupe cherry, 7–8 × 4–5 mm, stone oblong-conical, 5–6 × 2–2.5 mm.............................................................................*N. pamirica* L.I. Vassiljeva5. Drupe maroon to black in color, oval or spherical.................................................................7- Drupe yellow, orange, pale to bright red, oval, confined to Central Asia............................................6- Bush grows in Australia, leaves narrow at the apex, slightly pointed, stone conically ovoid.........*N. billardierei* DC.6. Stems ascending, leaves greenish-yellow, narrow, long, linear spatulate, 25–28 mm long, 2–3.5 mm wide; petals yellowish-white, ovate with short claws; number of flowers per inflorescence 17–30; drupe oval, 8–12 × 7–11 mm; stone conically ovoid with a recurved apex, 8.5–11 mm long, 4.5–6 mm wide; drupe sap light pink.......................................................................................................*N. komarovii* Iljin & Lava ex Bobrov7. Drupe black, oval or spherical; stone smaller than 7 mm..........................................................8- Drupe oval, maroon to black in color, 7–10 × 6–9 mm; stone ovoid, obtuse, 7–10 mm long, 4.5–6.5 mm wide; bush 0.7–1.5 m high, spreading-branching; stems arcuate, with large spines; leaves dark green, shiny, oblong-spatulate, 20–26 mm long, 3–6 mm wide; flowers yellowish-white in color; petals ovoid or diamond-shaped with short claws; number of flowers per inflorescence 11–28; fruit sap pale reddish................................................*N. schoberi* L.- Drupe oval, burgundy to dark purple in color, 8 × 6 mm; stone ovoid, obtuse, 6 × 3 mm; leaves oblong-lanceolate, 24–25 x 4.5–6 mm; bush 1–2 m high (up to 3–4)...................................................*N. tangutorum* Bobrov8. Leaves pubescent or slightly pubescent...................................................9- Leaves silvery from dense pubescence, calyx densely hairy, petals and ovaries hairy on the outside; leaf 10–15 × 4–6 mm..........................................................................*N. praevisa* Bobrov9. Bush 0.6–1.8 m high, dense, with small spines; leaves oblanceolate, elongated, green, 14–16 × 2–3 mm; number of flowers per inflorescence 40–80 (90); petals white, ovate or rhombic with short claws; drupe black, oval or spherical, 4–6 × 4–4.5 mm; stone narrowly ovoid with a narrow pointed apex; 3.5–5 mm long, 2–2.5 mm wide; fruit sap green-black.....................................................................................*N. iliensis* Banaev&Tomoshevich- Bush 0.2–0.8 m high, dense, with tightly arranged small spines and oblanceolate bluish-green leaves, 10–13 mm long, 2–3 mm wide; number of flowers per inflorescence 20–48; flowers white, pale-purple in buds; petals white, pointed-elliptical with narrow claws; drupe black, 4–9 × 4–8 mm; stone ovoid-acuminate, 3.6–6 mm long and 2.5–3.5 mm wide; fruit sap dark blue..............................................................................*N. sibirica* Pall.

## 3. Materials and Methods

### 3.1. Plant Material

**Figure 10:**
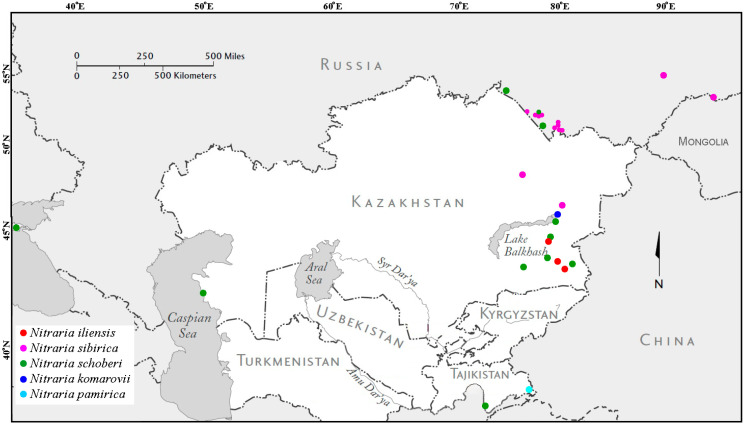
Distribution map of research specimens of the genus *Nitraria*. The collectors of all the samples indicated on the map are Banaev E.V. and Tomoshevich M.A.

The specimens of *N. sibirica* (11 habitats), *N. schoberi* (14 habitats), *N. komarovii*, *N. pamirica* and *N. iliensis* were collected in expeditions in Siberia (Novosibirsk region, Altai Territory, Republic of Tuva), Crimea, the Republics of Kazakhstan and Tajikistan in 2011–2017 (Figure 10). Field work was carried out in different seasons to observe species both in the flowering stage and in the fruiting stage. In each population, 25–30 herbarium leaves were collected (more than 800 herbarium specimens in total), and specimens of flowers, fruits, and seeds, which were packed in paper bags, marked and delivered to the laboratory of dendrology of the CSBG SB RAS (Novosibirsk, Russia) for morphometric analysis.

The specimens collected during the expeditions were deposited in the collection of the NSC CSSB SB RAS (Novosibirsk, Russia) and are available in the digital herbarium of the CSBG SB RAS “http://herb.csbg.nsc.ru:8081 (accessed on 22 December 2022)”. Sample voucher data are shown in Table 2.

Revision of herbarium materials was undertaken in the herbaria at LE, MW, NS, NSK, PE.

### 3.2. Morphological Analysis

The vegetative and reproductive morphology was studied on well-developed specimens of the generative age state. For numerical analysis, not less than 25 specimens were studied in each population of each species. Table 1 presents 26 morphological characters studied.

Morphological analysis was carried out using a Carl Zeiss Stereo Discovery V12 stereo microscope equipped with a high-resolution color digital camera AxioCam HRc and AxioVision 4.8 software for image acquisition, processing and analysis (Carl Zeiss Ltd., Göttingen, Germany), and the instrumental platform of the SIAMS Photolab image analysis system (SPF AVEK, 2013–2020) with the module morphometric analysis of plants.

Morphometric data were subjected to ANOVA using the STATISTICA 6.0 software (StatSoft Inc., Tulsa, OK, USA). The differences between means were tested for significance using the LSD test at *p* ≤ 0.05. In addition, clustering was performed with PCA. For PCA, relative metric parameters were additionally included: 4/3 is the ratio of leaf width to leaf length, 5/3 is the ratio of distance from the base to the widest point of the leaf blade to leaf length, and 24/25 is the ratio of stone length to stone width.

### 3.3. Palynological Analysis

For SEM examination, the air-dried pollen grains were dispersed evenly and put in double-sided adhesive-tape-covered aluminum stubs. The studs were coated with gold in a Mini SC 7620 sputter coater (Quorum Technologies, Laughton, Great Britain) and photographed under 20.0 kV voltage using the EVO MA10 (Carl Zeiss, Göttingen, Germany) scanning electron microscope.

About 25 pollen grains of each species were selected randomly, and the polar axis length (P) and equatorial axis length (E) of them were measured. All data were analyzed to calculate the mean (X), standard error (Sx), and the coefficient of variation (CV, %).

Palynological terminology was referenced from former works [56,57,58,59]. The pollen shape class, based on the P/E ratio, was identified using Erdtman’s system [60].

### 3.4. Molecular Analysis

Two spacer regions, one in the nuclear genome (ITS) and one in the plastid genome (trnH-psbA), were subjected to the molecular analysis. DNA was extracted from dried leaves using the conventional CTAB-based method [61]. The concentration and amount of the extracted DNA were evaluated in 0.8% agarose gel and using a spectrophotometer (NanoPhotometer P-Class, P-360, Implen, Munich, Germany). For amplification of different DNA sequences, a ready-made kit of GenePak^®^ PCR Core reagents (Laboratory Izogen, Moscow, Russia) was used. For amplification of the ITS operon, which includes intergenic spacers ITS1, ITS2, and the 5.8 s gene, primers ITS6 and ITS9 developed for East Asian species of the tribe *Spiraeeae* [62] were used. The amplification cycle included denaturation at 94 °C for 1 m, primer annealing at 58 °C for 50 s, and elongation at 72 °C for 1 m within 30 cycles.

For amplification of the trnH–psbA chloroplast locus, universal primers were used [63]. The PCR cycle included denaturation at 95 °C for 40 s, primer annealing at 59 °C for 50 s, and elongation at 72 °C for 90 s within 32 cycles. The quality of the obtained PCR fragments was verified in 1.5% agarose gel and purified with a kit for rapid DNA elution from agarose gels Diatom DNA Elution (Laboratory Izogen, Moscow, Russia).

Sequencing was performed in both directions for ITS and trnH-psbA at ZAO Evrogen, using an automatic analyzer model ABI PRISM 3500. Sequencing was performed using the BigDye Terminator v. 1.1 Cycle Sequencing Kit. The subsequent purification of products was performed using the BigDye XTerminator Purification Kit.

The sequences were viewed using Data Collection v. 3.1 and were read using Sequencing Analysis Software v.6. The sequences were pairwise-aligned using the BioEdit v.7.1.9 program [64], multiple alignment was performed using the ClustalW2 program, followed by verification of ambiguous positions in the chromatograms and manual editing. Aligned sequences were analyzed using the MEGA X program [65]. The phylogenetic tree was constructed using the UPGMA method and the maximum likelihood method based on the Tamura–Ney model [66]. To construct the phylogenetic tree, we employed fragments deposited in GenBankNCBI (*N. sphaerocarpa*: DQ267177; KP087761; *N. retusa*: KP087772; KP087764; *N. roborowskii*: DQ309042; KP087762; *N. tangutorum*: DQ267176, KP087763; *N. billardieri*: KP087775; KP087767; *N. komarovii*: KP087774; KP087766). *Peganum harmala* L. (KP087776; KP087768) was used as the outgroup closely related to the genus *Nitraria.*

### 3.5. HPLC-MS Analysis

Samples of air-dried and ground plant material (0.5 g leaves) were extracted with ethanol–water (50:50, *v*/*v*) in a water bath at 60–70 °C. The extract was purified using a C16 Diapack cartridge and dissolved in 70% ethanol. Mass spectrometric analysis was carried out at the Novosibirsk Institute of Organic Chemistry SB RAS (Novosibirsk, Russia). HPLC-MS analysis was performed using an Agilent 1200 liquid chromatograph (Agilent Technologies, USA) and a micrOTOF-Q hybrid quadrupole-time-of-flight mass spectrometer (Bruker, Germany) with API-ES. Positive ions were identified in the range of 100–3000 *m*/*z*. Chromatographic separation was carried out at 30 °C using a Zorbax SB-C18 column (2.1 mm × 150 mm, inner diameter 3.5 µm) with a ZorbaxSB-C8 guard column (2.1 mm × 12.5 mm, inner diameter 5 µm). The composition of the mobile phase changed in a linear gradient from 15:85 (*v*/*v*) methanol (phase A) and 2% formic acid in water (phase B) to 100:0 (*v*/*v*) in 30 min, then in isocratic mode from 30 to 45 min. The volume of the injected sample was 10 µL. UV detection was carried out at four wavelengths/bandwidths: 255/16, 270/16, 320/16, 340/32 nm. Mass detection operating parameters were as follows: dryer gas flow (nitrogen) 8 L/min, nitrogen temperature 230 °C, nebulizer pressure 1.6 bar.

## Figures and Tables

**Table 1:** Morphological characters of *Nitraria* species.

Number of Character	Character	*N. schoberi*	*N. sibirica*	*N. komarovii*	*N. pamirica*	*N. iliensis*
1	Height of bush (m)	0.7–1.5	0.2–0.8	0.5–1	0.1–0.3	0.6–1.8
2	Habit	Spreading-branching	Spreading-branching, dense	Branching, graceful	Spreading, dense	Spreading-branching, dense
3	Leaf length (mm)	20–26	10–13	25–28	13–17	14–16
4	Leaf width (mm)	3–6	2–4	2–3,5	2–3	2–3
5	Distance from the base to the widest point of the leaf blade (mm)	15–16	8–9	22	10	10–12
6	Leaf color	Dark-green, shiny	Glaucescent-green	Pale green	Green, matte	Green
7	Leaf shape	Oblanceolate	Oblanceolate	Narrow, linear-spathulate	Oblanceolate, with a pointed apex	Oblanceolate
8	Flowers	Yellowish-white	White (buds pale-violet)	Yellowish-white	White	White
9	Number of flowers per inflorescence	11–28	20–48	17–30	9–15	40–80
10	Flower spacing (mm)	0.4–0.8	0.2–0.3	0.3–0.7	0.2–0.3	0.3–0.5
11	Petal shape	Ovate or rhombic; claws short	Acuminate-elliptica; claws narrow	Ovate; claws short	Ovate; claws short	Ovate or rhombic; claws short
12	Petal length (mm)	3–4.7	2.6–4	1.7–3.9	2.1–2.3	3.5
13	Petal width (mm)	2–3.6	1–2.8	1–2.9	0.7–0.9	2.6
14	Anther length (mm)	1.2–1.4	0.6–0.9	1.3	-	0.8
15	Anther width (mm)	0.5–0.7	0.3–0.5	0.7	-	0.5–0.6
16	Pestle length (mm)	2.02–4.5	1.35–3.19	2.01–3.59	-	2.05
17	Pestle width (mm)	1.38–2.23	0.97–1.41	1.09–1.82	-	1.22
18	Fruit color	Dark-red to black	Black	Yellow, orange, or pale to bright red	Cherry red	Black
19	Fruit length (mm)	7–10	4–9	8–12	7–8	4–6
20	Fruit width (mm)	6–11	4–8	7–11	4–5	4–4.5
21	Weight of 100 fruits (g)	270–600	100–270	380–490	80–90	80–130
22	Fruit shape	Oval	Globate or oval	Oval	Oval	Globate or oval
23	Fruit sap	Pale reddish	Dark-blue	Pale pink	Pale pink	Black–green
24	Stone length (mm)	7–10	3,6–6	8,6–11	5–6	3.5–5
25	Stone width (mm)	4.5–6.5	2.5–3.5	4.7–6	2.0–2.5	2–2.6
26	Stone shape	Ovate, obtuse	Ovate, pointed	Conic-ovate with a pointed apex	Oblong-conical,	Narrow ovoid with a narrow pointed apex

**Table 2:** Vaucher specimens of *Nitraria* (Nitrariaceae).

No.	Taxon	Specimen	Locality	Date	Herbarium, Specimen Number
1	*N. sibirica*	Noven’koe	Russia, Altai Krai, Loktevsky District, vicinity of Noven’koe village	31 May 2011	NSK3001284
2	*N. sibirica*	Veseloyarsk	Russia, Altai Krai, Rubtsovsky District, vicinity of Veseloyarsk village	31 May 2011	NSK3001286
3	*N. sibirica*	Kulunda	Russia, Altai Krai, Slavgorodskiy District, on the shore of Lake Kulundinskoe	2 June 2011	NSK3001276
4	*N. sibirica*	Bele	Russia, Republic of Tyva, Tandinsky kozhuun, northern shore of Lake Bele	22 July 2011	NSK3001016
5	*N. sibirica*	Balansor	Russia, Altai Krai, Uglovskiy District, on the shore of Lake Balansor	1 June 2011	NSK3001280
6	*N. sibirica*	Dzhira	Russia, Altai Krai, Kulundinsky District, eastern shore of Lake Dzhira	2 June 2011	NSK3001283
7	*N. sibirica*	Gornyak	Russia, Altai Krai, Loktevsky District, vicinity of Gornyak village	31 May 2011	NSK3001289
8	*N. sibirica*	Kuchuk	Russia, Altai Krai, Blagoveshchensky District, vicinity of Nizhny Kuchuk village	2 June 2011	NSK3001274
9	*N. sibirica*	Yarovoe	Russia, Altai Krai, Tabunsky District, southern shore of Bolshoye Yarovoe Lake	3 June 2011	NSK3001282
10	*N. sibirica*	Shara-Nur	Russia, Tyva Republic, Tes-Khemsky kozhuun, the shore of Lake Shara-Nur	28 July 2011	NSK3000991
11	*N. sibirica*	Rubtsovsk	Russia, Altai Krai, vicinity of Rubtsovsk city	15 June 2013	NSK3001785
12	*N. schoberi*	Balhash	Republic of Kazakhstan, Almaty Region, on the shore of Lake Balkhash, sandy desert	31 May 2016	NSK3000948
13	*N. schoberi*	Sariozek	Republic of Kazakhstan, Almaty Region, 30 km north of Sariozek village	25 May 2016	NSK3000947
14	*N. schoberi*	Basshi	Republic of Kazakhstan, Almaty Region, vicinity of Basshi village	25 May 2016	NSK3000982
15	*N. schoberi*	Aidarli	Republic of Kazakhstan, Almaty Region, Zhambylskii District, 17 km south of Aidarli village	21 August 2017	NSK3000958
16	*N. schoberi*	Koktal	Republic of Kazakhstan, Almaty Region, vicinity of Koktal village	30 July 2013	NSK3000999
17	*N. schoberi*	Lepsi	Republic of Kazakhstan, Almaty region, on the bank of the Lepsi River in outskirts of Lepsi village	30 May 2016	NSK3000981
18	*N. schoberi*	Raz’ezd_47	Republic of Kazakhstan, Almaty region, vicinity of the village of Molaly (railroad junction No. 47)	15 August 2017	NSK3000944
19	*N. schoberi*	Bagan	Russia, Novosibirskaya Oblast’, southwest of the village Grushevka, on the terrace of Lake Bol’shoy Bagan	4 June 2011	NSK3000973
20	*N. schoberi*	Kaspii	Republic of Kazakhstan, Mangistauskaya Oblast’, vicinity of Aktau city, on sandy mound	12 June 2012	NSK3000979
21	*N. schoberi*	Actau	Republic of Kazakhstan, Mangistauskaya Oblast’, vicinity of Aktau city	12 June 2012	NSK3000978
22	*N. schoberi*	Krim	Crimea, on the sandy coast of the Black Sea in Dvuyakornaya Bay	16 September 2013	NSK3000960
23	*N. schoberi*	Pyandzh	Republic of Tajikistan, Gorno-Badakhshan Autonomous Region, on the sandy bank of the Pyandzh River	8 August 2014	NSK3000994
24	*N. schoberi*	Kulunda	Russia, Altai Krai, Slavgorodskiy District, on the shore of Lake Kulundinskoe	2 June 2011	NSK3000975
25	*N. schoberi*	Malinovoe	Russia, Altai Krai, Mikhailovskiy District, on the shore of Lake Malinovoe	1 June 2011	NSK3000971
26	*N. komarovii*	Balhash	Republic of Kazakhstan, Almaty Region, on the shore of Lake Balhash, sandy desert	31 May 2016	NSK3000920
27	*N. iliensis*	Basshi 1	Republic of Kazakhstan, Almaty Region, vicinity of Basshi village	25 May 2016	NSK3001499
28	*N. iliensis*	Basshi 2	Republic of Kazakhstan, Almaty Region, vicinity of Basshi village	30 July 2013	NSK3001277
29	*N. iliensis*	Taskarasu	Republic of Kazakhstan, Almaty Region, vicinity of Taskarasu village	26 May 2016	NSK3001244
30	*N. iliensis*	Karatal	Republic of Kazakhstan, Almaty Region, Karatalskii District, vicinity of Ushtobe city, on the terrace of the Karatal River	29 May 2016	NSK3000922
31	*N. pamirica*	Shaimak	Republic of Tajikistan, Gorno-Badakhshan Autonomous Region, Eastern Pamir, on the cliff of the Djilga River	10 August 2014	NSK3001238

**Table 3:** Sizes (μm) and shape of pollen grains of *Nitraria*. The data are presented as the mean (X), standard error (Sx), and the coefficient of variation (CV, %).

Taxa	Polar Axis (P)			Equatorial Axis (E)			P/E			Shape
	Range	X ± Sx	CV, %	Range	X ± Sx	CV, %	Range	X ± Sx	CV, %	
*N. sibirica*	36.11–43.33	40.24 ± 0.34	4.3	17.07–23.50	20.48 ± 0.30	7.5	1.73–2.28	1.97 ± 0.02	5.8	prolate
*N. komarovii*	23.38–29.50	27.15 ± 0.30	5.6	14.23–18.91	16.42 ± 0.30	9.1	1.44–1.89	1.66 ± 0.02	8.0	prolate
*N. schoberi*	38.19–49.90	42.84 ± 0.62	7.2	22.28–32.59	27.84 ± 0.65	11.0	1.24–2.05	1.57 ± 0.05	16.5	prolate
*N. pamirica*	30.13–36.07	32.29 ± 0.32	4.9	24.87–30.62	26.50 ± 0.28	5.3	1.18–1.27	1.22 ± 0.005	2.0	subprolate
*N. iliensis*	31.41–36.00	33.82 ± 0.32	4.8	21.10–27.08	25.81 ± 0.50	9.7	1.10–1.51	1.32 ± 0.02	6.7	subprolate or prolate

## Data Availability

Raw data are available upon request.
